# Understanding how and why health is integrated into foreign policy - a case study of *health is global*, *a UK Government Strategy 2008*–*2013*

**DOI:** 10.1186/1744-8603-9-24

**Published:** 2013-06-06

**Authors:** Michelle L Gagnon, Ronald Labonté

**Affiliations:** 1 Institute of Population Health, University of Ottawa, 1 Stewart Street, Ottawa, Ontario, K1N6N5, Canada; 2 Canada Research Chair, Globalization / Health Equity, Faculty of Medicine, Institute of Population Health, University of Ottawa, 1 Stewart Street, Ottawa, Ontario, K1N6N5, Canada

**Keywords:** Global health diplomacy, Health and foreign policy, Whole-of-government policymaking

## Abstract

**Background:**

Over the past decade, global health issues have become more prominent in foreign policies at the national level. The process to develop state level global health strategies is arguably a form of global health diplomacy (GHD). Despite an increase in the volume of secondary research and analysis in this area, little primary research, particularly that which draws directly on the perspectives of those involved in these processes, has been conducted. This study seeks to fill this knowledge gap through an empirical case study of *Health is Global*: *A UK Government Strategy 2008*–*2013*. It aims to build understanding about how and why health is integrated into foreign policy and derive lessons of potential relevance to other nations interested in developing whole-of-government global health strategies.

**Methods:**

The major element of the study consisted of an in-depth investigation and analysis of the UK global health strategy. Document analysis and twenty interviews were conducted. Data was organized and described using an adapted version of Walt and Gilson’s policy analysis triangle. A general inductive approach was used to identify themes in the data, which were then analysed and interpreted using Fidler’s health and foreign policy conceptualizations and Kingdon’s multiples streams model of the policymaking process.

**Results:**

The primary reason that the UK decided to focus more on global health is self-interest - to protect national and international security and economic interests. Investing in global health was also seen as a way to enhance the UK’s international reputation. A focus on global health to primarily benefit other nations and improve global health *per se* was a prevalent through weaker theme. A well organized, credible policy community played a critical role in the process and a policy entrepreneur with expertise in both international relations and health helped catalyze attention and action on global health when the time was right. Support from the Prime Minister and from the Foreign and Commonwealth Office was essential. The process to arrive at a government-wide strategy was complex and time-consuming, but also broke down silos. Significant negotiation and compromise were required from actors with widely varying perspectives on global health and conflicting priorities.

**Conclusions:**

As primarily an exploratory study, this research sheds significant light on the global health policymaking process at the level of the state. It provides a useful and important starting point for further hypothesis driven empirical research that focuses on the integration of health in foreign policy, how and why this happens and whether or not it makes an impact on improving global health.

## Background

Over the past decade, global health issues have become more prominent in foreign policies at the national level [1-6]. In 2007 the foreign ministers of Brazil, France, Indonesia, Norway, Senegal, South Africa and Thailand launched the Foreign Policy and Global Health (FPGH) initiative and the *Oslo Ministerial Declaration* and renewed it in 2010 [7,8]. Since 2008, the United Nations General Assembly has adopted three resolutions resolving that governments should pay more attention to global health in their foreign policies [9-11].

As nations become more interconnected and interdependent and health issues become increasingly global, state actors have more incentives to work together and with a variety of non-state actors on health issues that transcend national boundaries [12]. The process of negotiated collective action for global health has come to be referred to as ‘global health diplomacy’ (GHD), the ‘policy-shaping processes through which state, non-state and other institutional actors negotiate responses to health challenges, or utilise health concepts or mechanisms in policy-shaping and negotiation strategies, to achieve other political, economic or social objectives’ [[13], p. 10]. The manner in which this concept is used, however, is highly diverse and the GHD process itself poorly understood [13,14]. Little empirical research that draws directly on the perspectives and experiences of those involved in GHD processes has been undertaken leading to strong calls for more descriptive, analytical, conceptual and practical rigour [5,15-17]. This paper contributes to this goal by critically examining health in foreign policy (HiFP) through an empirical case study of *Health is Global Strategy*: *A UK Government Strategy 2008*–*2013* (*Health is Global*).

## Methods

The major element of the study consisted of an in-depth investigation and analysis of *Health is Global* launched in 2008. Of the countries with some formal strategy for GHD, the UK global health strategy is the most detailed and comprehensive.

**Figure 1:**
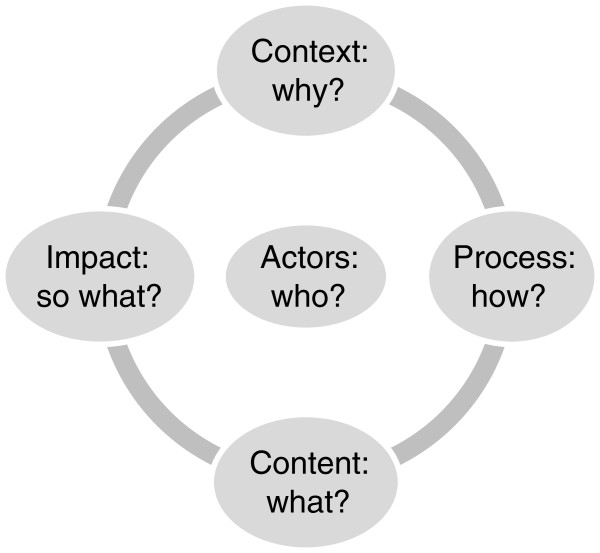
The Policy Analysis Circle.

Literature review, document analysis and semi-structured interviews were used to conduct the UK case, as well as three background case reviews (Norway, Switzerland and Brazil). This article reports only on the primary UK case. To structure data for subsequent analysis and interpretation using the theoretical frameworks (Fidler’s health and foreign policy conceptualizations and Kingdon’s multiples streams model of the policymaking process) an adapted version of Walt and Gilson’s policy analysis triangle [18,19] was used as an heuristic device to gather and organize a comprehensive and relevant set of data in five areas (Figure 1): the policy context within which the policy was developed (i.e. context for and

 reasons why the policy was developed); the policy processes (i.e. how the policy was developed and is being implemented); the policy content (i.e. the global health issues to be addressed through the policy and how health is positioned in the policy discourse); and the actors involved (i.e. who and what role they played in the process) [18,19]. A fifth important category, indications of impact, was added to capture data that focused on potential and actual effects of the policy.

**Figure 2:**
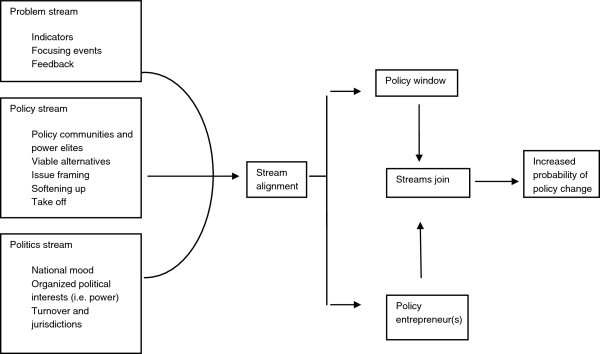
**A Multiple Streams Model of Policymaking [**[22]**].**

Data were analyzed, interpreted and explained using Fidler’s health and foreign policy conceptualizations [20] and Kingdon’s Multiple Streams Model of the policymaking process [21-23]. Fidler’s work is grounded in international relations theory and posits three arguments for why health has risen as a foreign policy issue – revolution, remediation and regression (Table 1). Kingdon’s model of the policymaking process is a highly reputable, evidence-based model that focuses on understanding why some topics become prominent on the policy agenda and others do not, and why certain policy alternatives are seriously considered while others are neglected (Figure 2). Together these frameworks provide a useful and novel mechanism for analyzing and interpreting the study findings and arriving at conclusions in light of the main research question – how and why is health integrated into foreign policy?

Purposive sampling was used to identify and recruit interviewees for semi-structured interviews. State and non-state actors who had been directly involved in health and foreign policy integration in each of the four countries were targeted for interviews. A total of twenty interviews were conducted, fourteen for the UK case (seven each with state and non-state actors). Access to interviewees was not an issue; however repeated

**Table 1:** **Summary of Fidler’s health and foreign policy conceptualizations**[20]

**Conceptualization**	**Description**
**Revolution**	• Health’s increasing role in foreign policy is transformative of the health-foreign policy nexus • Health collapses the traditional distinction between high and low politics and creates a new political space in which health is an overriding normative value and the ultimate goal of foreign policy • Health is broadly conceived and encompasses the social determinants of health • Is consistent with health discourses that focus on health as a human right and the “health for all” ideal
**Remediation**	• Health’s rise as foreign policy issue reflects the continued persistence of the traditional hierarchy of foreign policy functions • Health has become another issue that needs to be addressed through traditional approaches to foreign policy, or as a strategic vehicle through which traditional foreign policy goals can be achieved • Foreign policy attention on health is focused when disease crises appear and fades when crises drop off the political spotlight •Provides the strongest explanation for why health has risen as a foreign policy issue
**Regression**	• Health’s integration into foreign policy is a regressive development – an indicator that health problems are getting worse • The increasing attention paid to health across the functions of foreign policy signifies the failure of public health efforts • Connecting health with the high politics of foreign policy threatens to tarnish long-standing associations of health with normative values • Public health’s wish for health to become more politically prominent may have come true but in a way that threatens what was special about health in the first place

 attempts to recruit politicians who had been involved in the endorsement and approval of the UK strategy for an interview were not successful.

Interviews took place between August 27, 2009 and March 24, 2010. Six of the interviews with UK interviewees were conducted in person in London and the rest of the interviews were conducted by telephone. Interviews lasted from 30 minutes to 1.5 hours. Informed consent was obtained before the interview began. Interviews were audio-taped and then transcribed verbatim. Ethics approval was obtained from the University of Ottawa renewed on an annual basis until the study was completed and undertaken in full accordance with the University’s ethical guidelines.

A general inductive approach was used to analyze the interview data using manual coding [24]. Both the research objectives and questions (deductive) and multiple readings and interpretations of the raw data (inductive) guided data analysis. Data analysis encompassed three concurrent and iterative flows of activities: data reduction, data display and conclusion drawing/verification. The main themes in the data resulting from this process are reported on in the results section of this article within “why” and “how” categories consistent with the research question followed by a discussion and analysis of these themes using Fidler’s conceptualizations and Kingdon’s model.

## Results

*Health is Global* was released in 2008 largely in response to globalization and the realization that ‘the old distinction between ‘over here’ and ‘over there’ was becoming increasingly redundant’ and required nations to co-operate to achieve ‘health for all’ [1]. When released,

*Health is Global* was described as a ‘cross government strategy’ to highlight the breadth of challenges that face ‘all of us’ in the area of global health [25].

*Health is Global* is intended to span five years (2008–2013) however ‘its vision covers a 10-to 15-year period’ [1]. The strategy is comprised of goals with specific action areas and includes ten principles that are meant to guide decision-making particularly when conflicts among priorities arise. Sir Liam Donaldson, Chief Medical Officer for England and Dr. Nick Banatvala, Head of Global Affairs, UK Department of Health, acknowledged in their proposal for the strategy that potential conflicts exist between policy priorities.

‘For example, reconciling UK trade interests (including trade in commodities) with sound pro-poor development policy and maintenance of international human rights might be difficult…..A coherent UK global-health strategy is important in navigating an economically and ethically acceptable path through the priority areas’ [[26], p. 857].

The UK strategy’s final priorities and principles reflect the potentially conflicting reasons why it was developed and allude to the difficult process to reach a consensus on what eventually ended up in it.

In general, interviewees described the strategy as a very positive development referring to it as “motivational”, “a commitment to global health”, and “more than just another report”. Findings pertinent to how and why the strategy was developed follow, beginning with a brief overview of the British foreign policy from 1997 to 2008 that formed the backdrop to the strategy.

### Why?

The late 1990s marked the beginning of an increasing focus in the UK (and elsewhere) on the relationship between globalization and health with the UK’s Nuffield Trust playing a key role in catalyzing attention on this phenomenon and the importance of integrating health into foreign policy. The late 1990s also marked the beginning of Tony Blair’s premiership of the UK, a position that he held from May 1997 to June 2007 after which Gordon Brown became Prime Minister until 2010.

Three main foreign policies of Blair’s 10 year span as UK’s Prime Minister were an activist philosophy of international interventionism,^a^ maintaining strong alliances with the United States (US) and a commitment to placing Britain at the heart of Europe [[27], p.3]. Activist interventionism was regarded as a genuinely new perspective and approach, as was a focus on more ‘joined-up-government’ [27]. Under Brown, some ‘recalibration’ of the three themes occurred but there was more continuity than change from Blair to Brown [[27], p.3]. Of these three prongs, interventionism and the UK’s special relationship with the US appear to be the most relevant contextual factors that influenced why *Health is Global* was developed and what was included in it. A focus on more ‘joined up government’ also helps explain why *Health is Global* is a whole-of-government strategy.

#### The Blair Administration

During the 1999 Kosovo crisis Blair delivered his famous Chicago speech in which he unveiled his ‘doctrine of international community’ [27]. This doctrine was based on the explicit recognition that nations were becoming interdependent and that national interest was to a significant extent governed by international collaboration. Mutual dependence was linked to the idea that boundaries between the domestic and the foreign were blurring; therefore, an overriding policy of non-intervention was no longer an option. Indeed, in cases of genocide or crimes against humanity, it was a moral imperative.

While initially expressed through a paradigm of humanitarian intervention, after 9/11, Blair’s support for interventionism became linked with protecting national security, fighting terrorism and backing the US invasion of Iraq based on evidence about weapons of mass destruction in Iraq that it now appears Blair knew to have been fabricated [28]. This shift led critics to claim that respect for human rights and international law were subordinated to the UK’s focus on its relationship with the US and the ‘war on terror.’ Blair gave more attention to international development in his second term, which some argue was an effort to improve Britain’s tarnished reputation post Iraq [27]. One of the UK interviewees argued that *Health is Global* was in part politically motivated to the same end:

“*I think there was also*, *and I don*’*t know how much this motivated the government*, *but I think because of the opprobrium and the criticism of the UK government*’*s positions on the Iraq war and so on*, *I don*’*t know to what extent that might have influenced them to try and see how they might get a better international profile by focusing on positive contributions the UK could make to strengthening health and development*”.

A 2006 commentary published in *The Lancet* highly critical of the UK’s involvement in Iraq argued that ‘a renewed foreign policy that might at least be one positive legacy of our misadventure in Iraq’ [[29], p. 1396] was desperately needed. It concluded that health ‘is now the most important foreign policy issue of our time and should be used as an instrument of foreign policy’ [[29], p. 1397]. How much this article or the perspective it conveys influenced UK policymakers is not known. Its arguments, however, are explicitly referred to in the proposal that led to *Health is Global*, albeit without making any reference to the UK’s role in Iraq as a contributing factor [26].

The UK’s special relationship with the US may also have sparked *Health is Global* ‘s development. The US Institute of Medicine 1997 report, *America*’*s Vital Interest in Global Health*, is listed as a key influence and rationale for a government-wide strategy in the proposal that led to the strategy. The US report ‘identified three pillars: protecting people, enhancing the economy, and advancing international interests’ [[26], p. 857], which eventually became the thematic backbone of *Health is Global*. One of the interviewees also indicated that the Nuffield Trust’s relationship with US colleagues in the American Association of Academic Health Centers in the early 1990s was another key development that attracted more focused UK attention on the links between globalization and health and the relationship between health and foreign policy. As this interviewee explained, “*Basically the Association wanted to know whether we would have a continuing interest in working with the Americans on matters of mutual interest and on the health agenda*”. Stemming from this initial discussion, the Nuffield Trust began collaborating with the US on efforts to better understand the impact of globalization on health, which led to attempts to attract UK attention on this issue as well.

While a more sophisticated understanding of global health appeared to emerge during the Blair years, this did not necessarily translate into action [30]. Public health was not a dominant force in driving policy in the UK and the overriding reason for focusing on health in foreign policy was to protect national interest. Narrowly focused domestic security concerns were key motivating factors as seen in the emphasis given to bio-terrorism and infectious diseases [30].

#### The Brown Administration

Gordon Brown’s government retained the broad principle of interventionism but recast it to be less about hard power and more about conflict prevention and humanitarian agendas. He could not, however, completely repudiate the exercise of military power at a time when British troops were in both Afghanistan and Iraq, but he did emphasize that military action in the future would be a last resort [27]. In his speech to the Lord Mayor’s banquet on November 12, 2007, Brown summarized his approach as ‘hard-headed internationalism’ [[31], p. 15].

‘…internationalist because global challenges need global solutions and nations must cooperate across borders-often with hard-headed intervention-to give expression to our shared interests and shared values; hard-headed because we will not shirk from the difficult long term decisions and because only through reform of our international rules and institutions will we achieve concrete, on-the-ground results’ [[31], pp.15-16] [32].

Brown made it clear that the government’s primary obligation is the safety of the British people and the protection of the British national interests that would, in an interdependent world, be best realized through cooperation to overcome shared challenges [[31], p.15]. Many of these fundamental policy themes and the reasons behind them (economic globalization) permeate *Health is Global*.

In addition to traditional ‘high politics’ priority areas and partners (the US), the Brown government continued to promote international development. Over the course of the Blair to Brown years, the Department for International Development (DFID) enjoyed a reputation as a progressive, innovative and effective donor agency with a strong voice across government [33]. During that time, British aid spending tripled in real terms and it plans to spend 0.7% of gross national income on international development by 2013 [1,33]. Brown himself assumed a key leadership role in development efforts, including those focused on health. In July 2007, he launched the Millennium Development Goals (MDG) Call to Action along with the UN Secretary General [1] and in September 2007 the International Health Partnership, a global compact for achieving the health related MDGs [34]. Brown was a strong proponent of *Health is Global*. In the July 2009 Department of International Development (DFID)’s white paper he reiterated that even in tough economic times, ‘securing global justice remains one of my top priorities,’ [[35], p. 5] and recommitted to keeping the UK’s promise to support the realization of the MDGs.

The seeds for *Health is Global* were largely sown during the Blair years but it was under Brown’s leadership that the policy was launched.

#### ‘Economic prosperity, security and stability for the UK and the rest of the world’

The ultimate goal of the strategy is actually not global health *per se* but rather ‘economic prosperity, security and stability for the UK and the rest of the world’ [1]. As it states:

‘a healthy population is fundamental to prosperity, security and stability - a cornerstone of economic growth and social development. In contrast, poor health does more than damage the economic and political viability of any one country - it is a threat to the economic and political interests of all countries’ [[1], p.7].

Based on this reasoning it appears that global health is a means to an end and not an end in itself. Therefore, ‘improvements in the health of the UK and world’s population’ through ‘greater coherence and consistency between international policies that affect global health’ [1] are sub-objectives that support the overriding goal of economic prosperity, security and stability - traditional preoccupations of foreign policymakers.

#### Globalization

Several interviewees referred to the recognition of the important linkage between globalization and health as the driving force behind the attention it garnered from the Foreign and Commonwealth Office (FCO) in the early millennium. “FCO was a key player in late 1990s/early 2000s in the context of globalization”. “Globalization” required a “rethinking of how government works”, one in which “you need a joined-up approach”. This ‘joined-up’ approach had already been established “under the New Labour quite early on”. Another interviewee said that the strategy development process:

“…*looked at the whole issue and within that it became clear that globalization had an important linkage with global health such as communicable disease*”.

As stated in the strategy, ‘safeguarding good health is not simply the province of individual countries. A globalised, interdependent world, characterized by the increasing movement of individual and populations - and where disease recognizes no borders - means that health has become a global issue’ [[1], p.7]. But, as this same interviewee added:

“…*there were* [*also*] *opportunities clearly in healthcare as a growth area in terms of business opportunities*”.

#### “First it’s UK”

“*I think it would be foolish not to admit that a large part of it is done for UK benefit and that it has been recognized that there are global threats*. *So first it*’*s UK but longer*-*term benefits in terms of relationships and protection from threats and so on*. *There is a need and that runs through the development concept that it*’*s about working with other countries to reduce the global risk*. *The UK would want to protect its own positions*, *its own population*, *by recognizing these global threats*”.

The most prevalent and strongest rationale for the development of *Health is Global* is to benefit the UK. This rationale is evident through the focus on global health security (i.e. protecting the UK population from global health threats) that permeates the strategy.

In the wake of the 2003 SARS pandemic, the need to strengthen global health security and ‘ensure the safety’ [[1], p.3] of the UK population described as the ‘first duty of any government’ [[1], p.3] was clearly a strong, if not the strongest, rationale behind the development of *Health is Global*. As one interviewee put it, “we are united when it comes to being secure in the UK”. This focus is also a priority of the UK’s first ever national security strategy also launched in 2008 [36]. There is meant to be a ‘strong link’ [[1], p.15] between *Health is Global* and the national security strategy that includes the risk to the UK of diseases such as pandemic influenza along with international terrorism, weapons of mass destruction, conflicts and failed states [36,37].

While ‘global health security’ *per se* is not clearly defined in the strategy, findings from the interviews support global health security as the driving force behind the strategy. As one interviewee stated, “it does rather focus on diseases crossing borders which is probably one of the reasons it’s come to such a high profile”. Others noted that “One of the things that we’ve done in the UK is essentially accepted global health as the securitization of the health agenda”. There was some support for this perspective: “Development of sources or pockets of insecurity has led to, from my perspective, an equivocation of global health to global health security”.

One interviewee talked at length about how “through securitization health diplomats got into rooms that they weren’t previously in”. He described this as “piggybacking” on the securitization agenda to bring focus to global health issues more generally.

“*They* (*academic researchers*) *got invited to cabinet committees to sit at tables with four*-*star generals in a way that they weren*’*t able to previously*- *academic researchers suddenly found that they could advocate for research funding because they were talking about things that might kill millions of people*, *like AIDS*”.

While the majority of interviewees acknowledged that global health security was the main motivating factor behind the strategy, several of the non-state interviewees were highly critical of this positioning. One stated:

“*I know why they*’*re doing it*, *for government buy*-*in*, *but it*’*s not enough to think of health as a foreign policy as global security*. *With this global security thing you get governments who are in it for themselves*”.

Another commented that:

“*The security of health agenda has gone unchecked and unchallenged because too many people have too much to gain from it*. *I*’*m not saying it*’*s a bad thing but I*’*m not sure it*’*s not the great thing that we*’*re making it out to be*”.

These responses highlight a theme related to global health security, namely its potentially uneasy and conflicting relationship to global health equity. As one interviewee stated, “There are a lot of unaltruistic drivers of the development of the securitization of health agenda and one of these is the diminution of the health equity agenda”.

Although strengthening global health security, primarily as a way to keep the UK population safe, was the main rationale for its development, two other reasons were also cited (apart from the strategy functioning to deflect criticism from Blair’s Iraq debacle).

First, as one interviewee commented, the UK’s traditional “colonial” approach to foreign policy means it likes to be seen as a leader on the global stage and will do things in order to protect that reputation. Another interviewee stated that it was very “disappointing” that the UK did not sign onto the *Oslo Ministerial Declaration*. The comment was also made, “it’s so typical UK – have still got this old colonial, oh, we’re so great and think that we can go it alone”. In the same vein, “there was this thing that the UK still likes to see itself as a leader in things whether it is or not. That we must lead in global health. So the UK will do things in order to lead”. The proposal for the government-wide strategy also alludes to UK leadership as one of the driving forces behind it, ‘the UK has been at the forefront of multilateral initiatives, such as cancelling the debt for poor countries, access to medicines…the 2005 UK presidency of the Group of 8 wealthiest nations (G8) drew attention to global health, climate change, investment in health systems, and partnerships with government of developing countries’ [[26], p. 859]. *Health is Global* was seen as a logical extension of the UK’s leadership in global health.

Second, the strategy was developed in part to enhance UK business opportunities overseas in the context of globalization. ‘Health as a commodity’ was identified as one of the main reasons for developing the government-wide strategy in 2007 [[26], p. 858]. Indeed, harnessing ‘the force of globalization’ is largely about trade and investment opportunities for the UK, although in doing so it is also regarded as a way to improve global health and access to care and services for the ‘poorest people in the world [[26], p. 858]. One interviewee commented that, “you have UK companies looking to win business overseas” and another who said “there were opportunities clearly in healthcare as a growth area in terms of business opportunities”. The strategy seeks to enhance ‘the UK as a market leader in well-being, health services and medical products (including pharmaceutical and medical devices)’ including the priority to promote the ‘best of British healthcare’ both because it can contribute to strengthening health systems in other countries and also because it can bring ‘significant benefits for the UK economy’ [[1], p.29] [[38], pp.66-67].

#### For the benefit of others

Other rationales focused more on contributing to improving health and prosperity outside the UK as a goal in its own right. As stated in the foreword by the Prime Minister, ‘the strategy is one way for us in Britain to build a stronger, fairer world’ [[1], p.3]. Along with global health being a question of security, it is also a question of ‘morality’ and is defined in the foreword as a ‘force for good’ [[1], p.3].

As several interviewees described, *Health is Global* stems in part from the UK’s focus on development that became a more prominent part of the government’s agenda under Tony Blair. A separate Department of International Development (DFID) was created in the late 1990s and several policies that focused on the UK’s role in international development were released [38]. “There was a genuine interest in development in the government in the late 1990s and a growing concern about inequalities”, said one interviewee. The strategy itself comments that ‘improving health and reducing health inequalities requires tackling the underlying causes of ill health- the conditions in which people live and inequalities in the resources and opportunities to which they have access’ [[1], p.19]. It commits the UK to working with ‘the WHO, the EU and others to take forward key recommendations from the WHO Commission on Social Determinants of Health and ensure that actions to address these issues remains high on the international agenda’ [[1], p.19].

A few interviewees frequently linked the concept of “health equity” with that of “development”. As one noted, “In the UK we talk about development rather than equity”. Another commented, “I don’t see equity being a central concept in the policy discourse. It’s in a larger concept of development, which is then unpacked in various ways but I think very much informed by the neo-liberal premise”.^b^ One interviewee noted that the “equity lens is not fundamental. It’s just part of the discourse - part of the mix”, which another noted was placed and kept on the agenda by non-state actors: “activists, essentially, of one sort or another”. These comments downplay the importance of the health equity argument and are interesting because promoting health equity and reducing health inequalities is a fairly prominent concept throughout the strategy. One of the strategy’s ten principles explicitly refers to the importance of promoting equity within and between countries [1]. As well, health impact assessments are included in the strategy as a recommended approach to assessing the equity impact of domestic and foreign policy [38]. The inclusion of health equity as a priority in the strategy may well reflect the work and determination of a few strong non-state actors in the policymaking process. As one non-state interview noted, “I had to fight so hard to get human rights and health equity in it. They only play this card when it suits them”. This interviewee expanded further on this viewpoint, “they don’t really believe in equity either. Again it’s good when they want to score brownie points or something but if it means they are going to have to give over sacrificing something they’re not interested”.

While some interviewees expressed doubt as to the strategy’s commitment to global health equity, *Health is Global* nonetheless commits to investing in development. It aims to complement and build on DFID strategies by including actions focused on combating poverty and health inequalities in support of the MDGs and improving the social determinants of health in impoverished nations [1]. One interviewee praised the government’s commitment to development saying, “I think the British government … has been very proactive … partly because civil society [was] onto it straight away, in saying we will keep our overseas development commitments”.

Part of the rationale behind supporting development for health is based on the premise that ‘a healthy population is fundamental to prosperity, security and stability’ [[1], p.14]. Quoting the WHO Commission on Macroeconomics and Health (2001), the strategy reiterates that ‘ill health is a drain on society, while good health is a cornerstone of economic growth and social development in developing countries’ [[1], p. 14]. Taking this one step further, the strategy also asserts that in the context of globalization, poor health ‘does more than damage the economic and political variability of any one country - it is a threat to the economic and political interests of all countries’ [[1], p. 7]. While the development rationale is primarily about what the UK can do to help developing nations through trade and economic growth, it also includes elements of self-interest. As one stated, “security is now more centrally part of it (i.e. the reason for investing in global health)”, not development.

Human rights also figure in the strategy and its development. Donaldson argued that one of the reasons the UK must engage with the global health agenda through the establishment of a coherent global health strategy is because ‘health is a human right’ [39]. The right to health underpins the ten principles included in *Health is Global*. The strategy highlights that the UK was one of the original 1948 signatories of the Universal Declaration of Human Rights but does not make any additional references to its specific obligations under international human rights covenants. Having said this, *Health is Global* does commit to including health as a section in the government’s annual human rights report [1]. It also makes some explicit references to human rights with an emphasis on gender rights in the context of sexual and reproductive health and cautions that unfair or unethical trade can deprive workers of their ‘rights to security of employment and compensation’ [[1], p.60].

Of the 14 UK interviewees interviewed, five did not make any reference to human rights, and only two mentioned international human rights frameworks^c^ in their response. The majority of those who referred to human rights did so simply to affirm that human rights had been a consideration in the development of *Health is Global*. These comments embody the normative but not the legal dimensions of international human rights, which is consistent with how state actors tend to regard human rights as a rationale for focusing on health in foreign policy [37,40]. Only one interviewee explicitly referred to health as a “right” while others referred to concepts related to human rights such social justice and improving global health as an obligation. A prominent theme in the interview data was challenges associated with ensuring that a human rights perspective had an equal seat at the table in policy discussions along with trade, economic growth and security.

#### Influences from outside government

The Nuffield Trust played a key role in bringing the issue of the effects of globalization on health and HiFP to UK policymakers’ attention beginning in the later 1990s and early 2000s. As one interviewee explained, “the idea came a long time ago from outside government”, in particular from the Nuffield Trust and “from people with academic interest in global health diplomacy and the emerging concept of global health diplomacy”. The Nuffield Trust funded leading scholars to generate critical academic research as evidence in this area and used its position to build extensive networks of senior level officials engaged in health as a foreign policy issue [41].

Other members of civil society also appeared to play an influencing role in establishing the need for a greater focus on global health by state policymakers. As one interviewee commented, “I think civil society has definitely had an influence through campaigns like Make Poverty History”. The UK had played a leading role in launching the Make Poverty History campaign in 2005 which challenged the 2005 G8 Summit in Gleneagles to tackle issues of trade, aid and debt [42].

Developments in the international community that focused on health and foreign policy, in particular the publication of the *Oslo Ministerial Declaration* in 2007 also played a role. As one interviewee stated, these developments exerted “international pressure” on the UK “to get in the game”, though as already noted, and as this interviewee emphasized, “the UK likes to go it alone”. Another interviewee reflected:

“*So you have countries putting their stamp on their field saying this is what we understand by it*. *To me that raises the significance of a policy area*, *that when more than one state starts to do it then it becomes important for the UK to have its version of this discourse because it*’*s achieving a degree of international prominence*”.

#### Influences from inside government

The intent to put in place a whole-of-government approach to addressing global health was a major force behind its development. As one interviewee put it, “another thing to bear in mind with New Labour is the greater focus on government coherence, joined-up policies. It’s also important to see this as a driver for looking at how one policy area can impact on another”. As the strategy states:

‘Many UK government departments and agencies work on issues that directly or indirectly affect the health of the world’s population. To be most effective in our work on global health, and to make the most of opportunities to improve UK health, we need a consistent and joined-up approach across government’ [[1] p. 15].

An important factor that influenced and enabled the development of the strategy was the political support it had from the Prime Minister, Gordon Brown, and his Ministers of the day. Brown signed the foreword demonstrating “support from number 10”. Another interviewee reflected that Brown likely supported the strategy out of personal conviction:

“…*he*’*s committed the government to getting up to the UN target of 0*.*7*% *of GDP*, *he*’*s created this new financial vehicle for vaccinations and immunizations so he himself would seem very supportive of global health but has that been done for foreign policy reasons or because it happens to be his personal conviction*? *I don*’*t think he*’*s doing this in a major way for foreign policy objectives but out of personal conviction*”.

Ministerial support for the strategy reflected in a common voice and position across government was also critical and appears to have been a significant enabling factor leading to its development and eventual launch. Ministers that were the leads on collaborating to develop the strategy were present at its launch. The press release that accompanied the launch included quotes from each of them [25,43]. This demonstrated as one interviewee put it, “that the baseline was all signed up to this. That is why we have an HMG (Her Majesty’s Government) document”. Another reflected that:

“…*one of the things that I*’*ve learned working in government is that conducive personalities are the biggest driver for change*. *One minister getting on with another minister across the pond will do more for catalyzing or evolving a policy area or an agreement between countries than years and years of careful negotiation and planning*”.

As several interviewees communicated, the Foreign and Commonwealth Office (FCO) was a key player in the late 1990s/early 2000s in bringing attention across government to the rising significance of health in foreign policy. It also played a major role in ensuring that *Health is Global* was developed and launched. “FCO ran a series of workshops on a kind of interface of health and foreign policy that helped open a few doors to the strategy actually being published, to get the conversation going with FCO at an institutional level”. Several interviewees noted that “FCO support was key” and that there was a “push within government from a powerful part of government - FCO - to see this delivered”.

“*It would have been difficult to have seen this thing delivered if it simply came from the Department of Health*. *The strategy was led principally by the Foreign and Commonwealth Office*. *They discussed this in the context of globalization and how the UK should respond to it and there was agreement from that that one of the deliverables could be setting out what our global health policy*-*strategic approach could be and this dovetailed very nicely with what people were saying on the outside*”.

Several interviewees commented that there would not be a strategy without the lead public servant, Dr. Nick Banatvala, in the Department of Health (DH) who kept it moving forward. “There was a very, very committed individual in international health who was a dynamo, very, very brilliant and even when the time is right if you don’t have an individual, a sort of champion, then sometimes you don’t get things done”. Dr. Banatvala was described as the “real hero”. His understanding of the NGO world from which he came and his previous work with DFID were seen as critical to his success. He was also a medical doctor. It appears that Dr. Banatvala was successful in moving the strategy along not only because he was from the bureaucracy where “it really happens” but also because he had experience in and understanding of the different worlds, players and issues that needed to be integrated into the strategy. Another very important support for the lead public servant was “having people outside giving him the leverage to help inside government and for networking”.

### How?

#### Policy development process

In 2007, The Prime Minister and Cabinet approved Donaldson’s *Health is Global*: *Proposals for a UK Government*-*wide Strategy* discussion paper, which set out the rationale for a strategic framework for global health [[1] p. 15]. The DH led an interministerial working group that coordinated the development of the strategy, and in July 2007, several government departments, including FCO, DFID and Defense, and devolved administrations joined forces with *The Lancet*, the London School of Hygiene and Tropical Medicine and the Royal College of Surgeons of Edinburgh to host workshops with a wide range of stakeholders to debate what the UK government strategy should say [44]. Those developing the strategy also received written responses captured through the *Health is Global* website and reviewed commentaries published in health and medical journals about health and foreign policy. The results of the stakeholder workshop discussion were also published on the website and used to help shape the strategy. The interministerial working group for *Health is Global* oversaw the organization of these workshops, which aimed to involve the UKs devolved administrations in the process and a wide range of stakeholders from private, public and civil sectors, including those from the healthcare system, health insurance industry, academic and research organizations, the media, global health charities, health professional associations and advocacy groups [44].

Interviewees described the strategy development process as “an extensive exercise of consulting and getting feedback” that took about two years to complete. “It was clear how vast the agenda was”, said another. The process included a “cross-government priority mapping” exercise that “helped crystallize who was coming from what perspective”. As one non-state interviewee commented, “a lot of us learned a lot about how government works and in a way just that process itself was an important outcome. We got to know each other’s business”.

In addition to the development of background papers and stakeholder consultations, the policy development process also considered relevant research evidence. This evidence focused on the major causes of death and ill health in the world using data from the 2006 Global Burden of Disease and Risk Factors study and the 2006 Disease Control Priorities in Developing Countries report (DCP2) [38]. The findings from both reports appear to have informed a number of objectives and action areas in the strategy, including ensuring stronger, fairer and safer systems to deliver health and related actions such as focusing on non-communicable disease and injuries and identifying and supporting research and innovation that tackle global health priorities. The strategy also refers to both peer and non-peer reviewed literature and findings of important and relevant commissions such as the WHO Commission on the Social Determinants of Health, the WHO Commission on Macroeconomics and Health and the Codex Alimentarius Commission. One might conclude from this that there was significant attention paid to research evidence in the development of the strategy and in the final product. Interviewees, particularly those from the academic community and research organizations, tell a different story. While these interviewees acknowledged that there were deliberate efforts to involve academics and other sorts of researchers in the process because it was recognized that “there needed to be more evidence”, evidence was only one of many factors considered in strategy deliberations alongside politics, ideology and values.

“*The drivers are not necessarily that you*’*ve got a body of evidence why global health is important*. *Globalization is changing the context of health and that*’*s a general body of evidence*. *There*’*s a political and discursive element to this as much as an evidence*-*based one*. *It will always be couched as evidence based because that is the main legitimating discourse for policy innovation in the UK*”.

One interviewee stated, “My personal take is that there’s kind of a political rationale that’s important in understanding why this has happened rather than [it] being evidence based. To the extent that it is evidence based, its evidence of emerging infectious diseases”. Another commented:

“*How do you start thinking about evidence based policy for trade*, *for example*, *when it is such a political topic*? *I mean there*’*s an evidence base for pandemics because they*’*re the more scientific things but its other things even the climate change stuff that we*’*re just starting to do*. *So a lot of it is based on consensus*, *not evidence*”.

This particular interviewee also reflected on how evidence could be used going forward as the policy is being implemented:

“*My own ideal would be to have evidence collated now to develop policy further as well as supporting policy that exists*…*to be honest*, *it* … *could have done so much more in that section about the research and how research would be used to improve policy for the future and give a state of the art* – *where we are now and where do we need to get to*”.

In contrast to perspectives provided from researchers, one of the lead public servants provided another point of view:

“*At one stage we had quite a difficult time with some of those researchers because they felt that the document as a sort of earlier iteration was not sufficiently evidence based and there lies a tension between policymakers and researchers*. ‘*You*’*re identifying these four priority areas*. *Where*’*s the evidence for that*?’ *There is time when you accept that you take the evidence as it is and you move forward on a particular piece of policy*”.

#### Reconciling differences

Developing *Health is Global* and agreeing on a final product required significant consensus building and reconciliation of differences and interests across the many players involved. Moreover, the government of the day had committed to seeing the strategy developed so a “certain degree of pragmatism” was required to ensure a final product was arrived at in a timely manner. Early on in the policy process it was acknowledged that there were potential conflicts between the priorities that were emerging and that there might be difficulty reconciling UK trade interests with sound development policy [26,45]. Indeed, enhancing the ability to reconcile differences across government in the area of global health through a whole-of-government approach was one of the reasons the policy was developed in the first place [39]. As a way to reduce policy conflicts, in the strategy the Department of Health committed to supporting other departments in preparing global health impact assessments of their foreign and domestic policies [1].

According to several of the interviewees, the process of developing the strategy did indeed advance understanding about global health and the reconciliation of potential differences in this domain:

“*I guess one of the useful things that*’*s come out of this is that we*’*ve been able to improve discussions between* …*across government on what the different elements and issues are that intersect global health and then try to iron out what has been*, *at times*, *glaring contradictions in policy positions*”.

The process of ironing out contradictions and differences was clearly not an easy one, with the majority of interviewees describing it as difficult and requiring significant compromise to arrive at a final document:

“*It*’*s tough because our government doesn*’*t think the same on anything and each department has its own priorities and mandates so trying to get something that all would sign*-*off on including the PM was a big challenge*. *So he* (*the lead from the Department of Health*) *took stuff out*”.

Another commented, “I think it shows a bit of a tussle that it had to settle in order to be written. It had to settle for a slightly narrower definition of health”. Others described a “push and pull” process, “huffing and puffing over drafts” and being involved in interactions that “weren’t altogether as productive as they might have been” resulting in a product that “wasn’t truly a joint production”. Another commented that the “broad tone is collegial and amicable but it’s too far to say it’s consensual, there were very definite trade-offs”.

Interviewees provided significant insight into the trade-offs as well as to the power struggles that took place among government players. As one interviewee noted, “DFID is like an NGO in government. The powerful are trade, industry, FCO”. In keeping with the traditional ‘high politics’ areas of foreign policy, this comment likely explains what priorities rose to the top and received the greatest profile in the strategy (security and trade) as compared to those that received less (social determinants of health, health as a human right).

Two main areas that required negotiation and compromise were clear in an analysis of the interview data. First, as one of the comments in the previous paragraph highlights, there was a lack of consensus as to what global health actually is. For example, there were those who regarded global health as primarily about diseases that cross borders (e.g. Health Protection Agency) and others who regarded it as being much broader and also encompassing the social determinants of health (e.g. Department of Health International Unit, DFID, NGO interviewees). As one interviewee explained:

“*The policy community that focuses on global health is very*, *very small*. *You*’*re basically talking about one unit of a unit within the Department of Health*. *I think not more than half a dozen middle*-*ranking civil servants in DFID*, *and I*’*d be surprised if we*’*ve got half a dozen people in FCO who have a specific portfolio brief for global health*. *To them global health means all the things that have been before*, *special health regulations*, *but also trade and IP*, *migration policy and health* – *outside that group of people*, *global health is equated to health security*”.

To resolve this issue, it appears that the players agreed to settle on what one interviewee called a “slightly narrower definition of health”, couched primarily within the rubric of global health security.

Second, as anticipated when the strategy was first under discussion, there were significant debates related to priorities that may conflict, such as ‘enhancing the UK as a market leader in well-being, health services and medical products’ on the one hand while ‘promoting access to medicines’ [1] on the other. In other words there were conflicts between priorities that would primarily benefit the UK and certain interests within the UK (e.g. trade, security) versus those that were meant to primarily benefit others (e.g. development, human rights). Several themes in the interview data elucidate this struggle further.

The first example has to do with international trade in conventional arms, which is a significant issue given the UK is one of the world’s largest arms exporters [37]. As an interviewee from FCO said:

“*There are certainly a lot of civil society organizations saying if you are serious about improving global health outcomes*, *you should be tackling the arms industry*. *Now that takes us into very*, *very sensitive territory for FCO because you know*, *automatically there are going to be conflicting interests at play*”.

A few other interviewees also commented on the arms issue with one indicating that “there were some pretty robust discussions between the Ministry of Defense, Department of Health and the Foreign Office around what our global health strategy would mean to things like arms agreements”. Another added:

“*I remember at one point we had a discussion of arms and how you know*, *how the arms industry was going to be integrated into all this and accepting that countries have a right to defend themselves but nevertheless*, *some arms exports end up in regimes which are unsavory*, *to say the least*. *I think that report rather dodged around that kind of issue*”.

This interviewee is likely referring to the section of the strategy in which the UK calls for a legally binding treaty for the international trade in conventional arms without impinging on ‘legitimate, responsible defense exports’ [[38], p. 21]. What this means exactly is not elaborated on in the strategy but it can be assumed that “dodging the issue” through lack of clarity and the use of diplomatic language was perhaps the only way that relevant government departments would collectively sign off on this content in the strategy.

Another concern that at least half of the interviewees mentioned relates to the issue of advancing UK as a market leader in health and supporting UK industries abroad while at the same time also aiming to reduce health inequalities through, for example, contributions to improving health systems and access to medicine and technology in low and middle income countries. While the previous example brought in FCO, Defense and Health, comments about this issue focused primarily on conflicting priorities across DFID and the Department of Health. As one interviewee stated, “part of the DH role is to act as a sponsor for the UK health economy and you’ve got Trade and Industry which are responsible for trade promotion. DFID has been working on access to medicines so you can infer a potential kind of conflict there”. Another elaborated further:

“*When you look at trade and intellectual property issues*, *DFID would always say*, *well*, *look*, *what can we do for the developing world*? *And when that comes into conflict with actually what might be most beneficial for UK companies in terms of how they can get stronger intellectual property protection globally*, *DFID will not soften its stance which would be at odds with what other departments are doing*, *say the Department of Health which is the lead sponsor department for the pharmaceutical industry within government and would want to pursue a policy position that government would be sympathetic to industry*”.

As an interviewee from DFID emphasized:

“*The thing that drives us and drives most development agencies are the MDGs*. *That*’*s our focus*, *that*’*s our mission*. *That will drive things first and then we will try to align with other domestic partners*. *Our first and foremost objective is reducing poverty*. *That comes before anything else*”.

One interviewee provided significant insight into the nature of the discussions that took place to hash out these sorts of conflicts and expressed some frustration with the DFID position. These comments also highlight that one of the positive aspects of going through the process of developing a strategy was the opportunity to hold discussions about contentious issues since it was imperative that a strategy be agreed upon and launched.

“*There were times when we got to air some intellectual laundry that we never*, *never got to in public or private between ourselves before*. *I remember one particular exchange when we wanted issues related to medical devices*, *the pharmaceutical industry*, *the biotechnology industry*, *wanted all three of those approaches to get into the chapter and so we sat down*, *we had a meeting and agreed to the points we wanted to make and I went away and produced a draft*. *We shared it with our colleagues in DFID and it*’*s not to say that there was ever any kind of intention of fighting but their comments displayed naiveté about the importance of economic issues and wealth generation for the UK and they*’*d state*, *which is true*, *our department can*’*t support priorities which are about the UK getting richer*. *And we*’*d say*, *well*, *we understand that this isn*’*t DFID speaking*, *this is the UK government*. *It*’*s part of the kind of mentality that happens with all governments*. *It was frustrating that certain departments and certain colleagues still didn*’*t make the intellectual leap required to have a joined*-*up piece*”.

When asked how these sorts of issue were eventually resolved so the strategy could be written, this interviewee said, “That particular chapter ended up being compromised and shrunk in size, unfortunately”. It also appears that there was an agreement between DFID and DH that the strategy would reiterate DFID’s commitment to working with ‘the poorest countries in the world’ [[38] p.58] while DH would concentrate on middle income and emerging countries, such as Brazil, India and China [1].

Another interviewee provided some additional thoughts on how tensions were resolved during the policy development process:

“*I think there was a group of people who have a particular priority focus but who fundamentally have the same values and therefore discussion and re*-*discussion and redrafting and ensuring that the text reflected the commitments of the department whichever department you came from was not a completely painless process but it was done in a number of iterations to ensure that all stakeholders were content*. *And I think that was a critical part of ensuring that the strategy itself was actually accepted*”.

Despite the contentious issues that arose during the process and trade-offs and compromises that were required to “all meet in the middle”, the majority of interviewees were satisfied with the final product. The process of developing it was seen as beneficial in achieving greater cross government understanding of issues and policy positions.

#### Policy implementation process

The strategy includes a detailed implementation plan with specific actions each with an assigned lead department(s). An interministerial group made up of representatives from the departments involved in the development of the strategy (DH, Defense, DFID, FCO) is responsible for implementing the strategy and monitoring progress [1]. A cross-government steering group of senior official supports the interministerial group [1]. Actions it is taking to ensure partner involvement include regular partner events to review global health challenges and to assess whether the strategy is making an impact [38].

The strategy did not commit new resources to support implementation but rather reiterated the relevant resources that it had already committed to global health, particularly those for international development funneled through DFID. The strategy also emphasized that existing resources from other government departments are important and that ‘these resources need to be used strategically if they are to have maximum impact. This means supporting the priorities and approaches set out in the strategy and working with others to deliver them’ [[1], p. 33].

One area of new investment included in the strategy pertains to the global health security priority. While details of the level of funding and which department is contributing to it are not provided, the strategy commits to ‘new funding for the HPA (Health Protection Agency) to do more work internationally’ [[1], p. 21] and support for a new Chatham House Centre on Global Health and Foreign Policy [38]. This investment demonstrates the importance of the strategy’s global health security priority. In addition, the strategy also commits to providing funding for the new European Council on Global Health that aims to strengthen the European voice in global health governance and be a powerful advocate for a sustainable European commitment to global health. At the time this study was undertaken, Global Health Europe Task Force members included Dr. Nick Banatvala, who led the development of *Health is Global*, and Dr. David Heymann, Director of the Centre for Global Health Security at Chatham House [1,46,47].

Several interviewees mentioned the role of Chatham House in helping to implement the strategy. Most considered this to be highly positive given its long standing reputation as a ‘world-leading source of independent analysis, informed debate and influential ideas’ about international and global issues [48]. One interviewee from an NGO, however, was highly critical of this move, arguing that Chatham House has no experience in health and its focus on global health security as opposed to health equity was a “cop-out”.

The *Health is Global* strategy set out a set of actions against which indicators would be developed and progress measured. It committed to reviewing progress regularly ‘to improve the way we are working,’ [[1], p. 16] and overall impact at the end of the life of the strategy to determine what to do next [1]. As part of the evaluation process, it would commission annual independent reviews on progress on particular aspects of the strategy with a full review in 2013. It is not clear if such reviews have indeed been annual as only one such review conducted in June 2010 is publicly available [49]. It does appear, however, that the interministerial group is tracking progress on a regular basis since the strategy was released, as reported at partner meetings held at Chatham House and in partner newsletters [50,51]. Furthermore, the UK government launched a *Health is Global* outcomes framework in 2011 [52]. Starting with the original strategy and the recommendations from the first independent review just mentioned, the government developed an outcomes framework to support the next phase of the strategy. This framework reaffirms the guiding principles and focuses efforts towards achieving a consolidated set of twelve high-level global health outcomes by 2015 that will be underpinned across government by departments’ own delivery plans.

Interviewees provided their perspectives both on what impact they thought the strategy has had so far as well as perspectives on success going forward. Overall, interviewees regardless of sector described the strategy as a positive and important milestone, particularly because it focused minds in a “more consistent way” across government, has been a “good driver” for individual and collective work because it is now “written down” and serves as a guide for identifying “how each department fits and where the gaps are”. It was also described as a concrete example of the UK’s commitment to global health, “sticking our flag in the sand is a successful output” said one. Several mentioned what they regarded as concrete positive outcomes of the strategy so far, including the launch of the research program at Chatham House and new funding for the Health Protection Agency. Another commented that the strategy is making a difference because it “builds awareness and support for the MDGs”. A few interviewees nonetheless remained querulous about the strategy’s impact:

“*Has the government kept to it*? *Is the government who signed onto it keeping to it*? *What has this strategy led to that would not have happened anyway*?”

And:

“*Success will be what happens to the policy community around this*. *Will there be greater interaction between FCO*, *DFID and Health*? *Greater cooperation*? *Genuine engagement*?”

## Discussion

### The importance of actors and leaders

Different types of actors played a significant role in influencing the creation of *Health is Global* and ensuring that it was developed, launched and implemented.

#### The policy community

While *Health is Global* was launched in 2008, the policy community had been actively influencing its eventual development for at least a decade earlier. The Nuffield Trust, in particular, played a major role in attracting and sustaining focus and analytical scrutiny on the link between globalization and health and, with partners, in connecting the various players in the policy community (e.g. government, academia, think tanks). This leading and connecting role is critical to preventing the fragmentation of the community and the policy alternatives it espouses, which can significantly weaken such a community’s clout as influencers in the process [21]. A more closely knit policy community can generate consistent ways of thinking, common language and issue framing, all of which are important to softening up a policy space and stabilizing a policy system to influence change. That *Health is Global* was framed according to recommendations stemming from the Nuffield Trust led processes indicates that this policy community had an impact.

Government actors are part of policy communities and in the UK case the most prominent of these were FCO, DH and DFID. Whether actors from these three sectors considered themselves to be part of the same policy community during the policy development process is not known, although given the significant consensus building that was required to arrive at an agreed upon strategy, likely they did not. Instead, as several interviewees described, the policy development process itself brought departments with disparate views closer together creating somewhat of a closer knit policy community in government.

An interesting observation stemming from the interview data pertains to the somewhat tense interactions that academics who contributed to the process had with government policymakers. On the one hand, academics thought that there needed to be a greater focus on gathering and scrutinizing evidence to inform the policy, while on the other, the policymakers were focused on being pragmatic and moving forward with whatever evidence they had on hand. This tension is not surprising and is supported by ample literature about the challenges associated with the evidence-informed policy and decision making processes [53-59].

It appears, then, that while there was representation and participation from the academic community in the *Health is Global* process this does not necessarily go hand in hand with the conclusion that research evidence played a central role in influencing policy decisions. Drawing on conclusions derived from the application of Kingdon’s model, policy is primarily the result of politics, policy entrepreneurs and the convergence of the three streams and not the result of research evidence *per se*. The interview data corroborates this conclusion. To repeat one particularly relevant comment, “my personal take is that there’s kind of a political rationale that’s important in understanding why this has happened rather than being evidence based. To the extent that it is evidence-based, it’s evidence of emerging infectious diseases”. This comment resonates with Labonté’s argument that technical evidence, especially about risk and pandemic preparedness, may have traction in global health policymaking as it aligns with the health security focus, but rarely is there a full consensus on evidence with respect to other global health areas such as aid and development, leading to a significant amount of political interpretation [60].

#### Policy entrepreneurs

According to Kingdon’s model, policy change cannot take place without leadership from tenacious policy entrepreneurs [21]. In the UK case a policy entrepreneur played the key leadership role in advancing policy directions. While such entrepreneurs do not necessarily need to be politicians or public servants, based on the findings from this study (including the three background cases not reported on in this article) leaders in GHD processes appear to possess at least two special attributes. First, they are either politicians or senior public servants, and second, they encompass both health and international relations expertise through formal training and/or education or a combination of the two. Three of the four leaders in the case studies (including the three background cases) were medical doctors who could call upon their status as the elite profession within health, as needed. Despite their authority and influence, however, policy entrepreneurs cannot be successful unless they have backing of those from the highest level of political power. In the UK case, Prime Minister Brown was personally committed to *Health is Global*. Support from “number 10” was viewed as essential for the process to succeed. Similar political support and policy leadership from the very top was seen as necessary for the policy directions taken in the background cases.

According to Kingdon, policy entrepreneurs play the key role in ‘softening up’ the system and linking the problem, policy and politics streams. One way in which they do this is by developing their ideas and proposals in advance of when a policy window may open. This was indeed what happened in the process leading up to *Health is Global*. “The real hero” as one interviewee called him, Dr. Bantavala, contributed to the precursor proposal, a summary of which was published in *The Lancet*[26].

#### World Health Organization (WHO)

At about the same time that the UK released *Health is Global*, it also published a *UK Institutional Strategy* that will guide and frame its work with the WHO. The UK’s WHO strategy is a joint strategy of the health, international development and foreign affairs departments. The strategy coheres with *Health is Global* and sets how the UK and WHO will work together most effectively to support the goals and objectives of the UK government and of the WHO [61]. This strategy and the multiple references that *Health is Global* makes to the priority that the UK places on working with and strengthening the WHO to advance global health objectives is consistent with findings from the background cases.

The UK WHO strategy acknowledges that as a ‘major force for good in global public health,’ [[61], p.6] the WHO is at the heart of responding to global health challenges, is responsible for providing leadership in global health matters and is also a key development partner for delivering on the MDGs [61]. The UK acknowledges that the WHO as an institutional actor in the context of globalization plays a major role in helping them to cooperate to achieve common global health objectives. While self-interest prevails as the main reason that states like the UK are developing strategic approaches to investing in global health, acknowledging that the WHO is an important and relevant actor, though also in need of significant reform [62-64], signals that negotiation and consensus building to improve population health both within and across states is both necessary and possible.

#### The importance of timing and stream alignment

Timing and the alignment of the problem, policy and politics streams found in Kingdon’s model were critical to the eventual development and government-wide agreement on *Health is Global*. The growing awareness of global health and the potentially important relationship between health and foreign policy (part of the policy stream) had been brewing for several years in the UK policy community before the SARS crisis hit as the “wake-up call” for the government to take concrete action. While SARS was what Kingdon would call the ‘focusing event,’ (a component of the problem stream) there also appeared to be political motivation - to improve the UK’s global reputation post Iraq. Investing in global health was arguably one potential way to do this. The UK’s commitment at the time to helping to achieve the MDGs (another aspect of the problem stream) was also a strong motivating factor for focusing on global health. Within this mix of policy, problems and politics, leaders within the bureaucracy (policy entrepreneurs) had set the stage for catalyzing stream alignment when the policy window opened with the SARS crisis. A bandwagon effect occurred at that point in time that and created incentives for the various government actors with non-state actor participation to arrive at an agreement on whole-of-government global health policy: the *Health is Global* strategy.

#### Revolution? remediation? regression? - self-interest dominates

Findings in the UK case lead to the conclusion that *Health is Global* was developed primarily to benefit the UK. Such self-interest is reflected in the strategy’s focus on global health security, the priority the strategy places on capitalizing on global health as a business opportunity and the revelation that the strategy was likely developed in part to improve the UK’s global reputation that had been tarnished as a result of its involvement in Iraq. These observations align with Fidler’s remediation conceptualization, that the strategy is using global health to further other traditional foreign policy goals. In contrast to the revolution conceptualization, health is not an overriding normative goal of foreign policy but rather a means to an end.

While “First it’s UK” was the driving motivation behind *Health is Global*, not all interviewees agreed with this rationale, arguing that it was a threat to health equity and undermined development efforts. Using development aid to further the UK’s security agenda is one of the policies that Britain’s new Prime Minister, David Cameron, appears to be supporting. In his first Lord Mayor speech in November 2010, Prime Minister Cameron, like Brown before him, focused on hard-headed internationalism albeit with an even stronger ‘hard-headed’ intent.

‘Our foreign policy is one of hard-headed internationalism. More commercial in enabling Britain to earn its way in the world, more strategic in its focus on meeting the new and emerging threats to our national security…. Above all, our foreign policy is more hard-headed in this respect. It will focus like a laser on defending and advancing Britain’s national interest’ [65].

This statement reinforces the conclusion that it is the primacy of self-interest that will drive foreign policy under the Cameron government, potentially even more so than it had under Brown. In October 2010, Cameron unveiled the new UK security strategy allocating a larger proportion of DFID’s budget to addressing issues of conflict [66]. Strengthening governance and security in fragile and conflict-affected countries, in particular Afghanistan and Pakistan, is among DFID’s five priorities [67]. Critics described this as ‘development as counter-terrorism’, arguing that aid should be disbursed on a needs basis and not ‘according to Whitehall’s security agenda’ [68].

Investing in development based on self-interest also appears to be part of the messaging in DFID’s 2011–2015 business plan which refers to development as ‘tremendous value for money and good for our economy, our safety, our health and our future ’ [[67], p.2]. In keeping with the government’s new structural reform agenda, the plan strongly focuses on demonstrating value for money with an emphasis on results, transparency and accountability. While this approach to aid can potentially allow a better assessment of aid effectiveness, carried to an extreme it could end up favouring projects with short-term deliverables at the expense of long-term infrastructure, or on countries with a greater existing capacity to show returns at the expense of more vulnerable states [37]. Having said this, and while DFID’s business plan contains certain ‘hard-headed’ elements, it also includes those that reflect the UK’s commitment to benefiting others by investing in aid. The plan reiterates the UK’s commitment to spending 0.7% of gross national income on aid by 2013, which OECD reports the country is well on the way to achieving, [69] and includes priorities such as leading international action to improve the lives of girls and women, combating climate change, responding to humanitarian disasters and improving the global development system [67].

While self-interest manifested through the global health security framing may attract the attention of foreign policymakers, such positioning is potentially fraught with risk for global health and can lead to what Fidler’s refers to as global health ‘regression.’ Health may have risen as a foreign policy concern but in a way that tarnishes it’s normative underpinning or what made health special in the first place leaving it at the margins of traditional foreign policy and vulnerable to shifting foreign policy attention. Adding to this risk is the lack of a clear and universal definition of global health security. As Aldis argues, policymakers in industrialized countries emphasize protection of their populations against external threats when talking about global health security, while policymakers in developing countries and the UN system understand the term in a broader public health or human security context [70]. This definitional problem may help explain why the term is used somewhat confusingly as a catch-all phrase in the *Oslo Ministerial Declaration*. As a policy position developed by Ministers from both developed and developing nations it raises the question as to whether there was a common understanding of this concept as presented in the *Declaration*. It is also difficult to assess the impact of such high profile framing on other global health policy processes, but as Kingdon’s model highlights, issue framing is an important part of the policy process that can lead to a significant bandwagon effect [21].

#### Policy process is global health diplomacy

As noted in the Introduction, GHD is generally considered to involve policy-shaping processes around health challenges, or utilizing health concepts and issues to achieve other political, economic or social objectives. The examination of the *Health is Global* policy process provides evidence to support this definition. It also leads to a few specific conclusions about the nature of GHD at the state level when actors aim to develop whole-of-government strategies. As a starting point, the in-depth analysis of the UK process allows a number of more specific defining characteristics to be formulated:

•While non-state actors provide important inputs into the process, the final negotiation of the content of the strategy takes place among state actors, in particular those representing health, foreign affairs and development government departments which, assuming that there is political will behind the policy direction, are compelled to arrive at a strategy within a given timeframe that is acceptable to all relevant government actors. As the UK case revealed and the Kingdon model helps to explain, the process leading up to the state negotiation stage can be lengthy, potentially lasting many years. It is during this time that non-state actors can act in policy communities as policy advocates softening up and framing the policy space. Connections with policymakers and other policy entrepreneurs provide opportunities to influence policy direction further as does the development of evidence to support varying policy alternatives. Non-state actors can play an important challenging function, particularly during the strategy framing process, by drawing attention to global health equity issues.

•State actors negotiate the finer details of the strategy but as the UK case showed a process that aims to include actors from the public, private and civil sectors who will work with government to eventually implement the strategy appears to be an effective approach. The desired outcome helps to determine what process to put in place. If the intent is to develop a comprehensive global health strategy that will require multi-sectoral actors to help implement such actors should be involved as partners in the process from the outset.

•Leadership in the policy process by an authoritative, credible policy entrepreneur is a critical success factor. Such leaders have specific attributes, the most important of which is that they have knowledge, experience and training in both health/medicine and international relations enabling them to understand, be credible and connect within both contexts. Political leadership from the head of government is also critical.

•The whole-of-government process is difficult, complex, fraught with differing policy perspectives and positions and time consuming. Skillful negotiation and consensus building is required to arrive at an acceptable strategy for all involved as are cross government processes and structures such as interministerial working groups and committees. The UK case showed that significant compromise could be required to reach an agreement and ‘sign-off’ on a strategy. While the process is difficult, interviewee comments indicated that it was nonetheless an important way of building common understanding across government and broke down silos to working together. This was perceived to be a positive consequence of the policy development process.

## Conclusions

This paper provides significant insight into why and how health is integrated in foreign policy, which has helped to better define and crystallize the global health diplomacy process at the state level. Many of the main conclusions are similar to the unreported findings from the three background cases conducted as part of this study. Self-interest is the dominant reason that the UK developed *Health is Global*, a rationale that could become even stronger and deeper in a climate of economic constraint. This conclusion is consistent with the results of the Norway and Swiss cases in which self-interest was also the dominant rationale for investing in global health, i.e. to protect national and international security and their economic interests. In these cases, consistent with that of the UK, investing in global health was also seen as a way to enhance the state’s international reputation. In terms of self-interest, however, Brazil was an outlier. International solidarity and health as a human right have been the driving forces behind its long-term investment in development cooperation to date. Investing in health for normative reasons was also a prevalent though weaker theme in the UK, Swiss and Norwegian cases.

In the UK case and the three background cases, the role that policy entrepreneur leaders, particularly those with expertise and experience in both health and international relations and other actors play in the process is extremely important. The WHO is regarded as a highly important and relevant institutional actor in global health diplomacy but recently it has been argued that organizational reforms are greatly needed if it is to continue to play this role effectively. This discussion also highlighted particular characteristics of the global health diplomacy process at the state level that may be helpful for other states to consider when developing similar whole-of-government global health strategy. Even if the current context in such countries is not ideal for such a strategy to take root because of the world’s current economic situation, based on a more in-depth understanding of the process, it is important for policy communities and entrepreneurs to remain persistent in their efforts to influence and lead policy change. The softening up process can take many years but is an important preparatory phase in creating readiness for policy change when the ‘problem, policy and politics’ streams align and a policy window opens.

## Endnotes

^**a**^Interventionism is defined as the use or threat of force or coercion to alter a political or cultural situation normally outside the intervener’s moral or political jurisdiction. It commonly deals with a government’s interventions in other governments’ affairs [71].

^b^By “neo-liberal premise” this interviewee was referring to an investment in development to further state economic interests in the context of globalization.

^c^ The right to health (technically, the Right to the Highest Attainable Standard of Physical and Mental Health) is the human rights statement most central to GHD, and every country is now party to at least one international instrument that includes health-related rights [72]. Article 12 of the International Covenant on Economic, Social and Cultural Rights (ICESCR) provides the most detail on this right [73]. General Comment 14 (GC 14), legally less binding than Article 12 itself, elaborates a broader range of actions required for the progressive realization of this right [74]. GC 14 further states that ‘collective rights are critical in the field of health’ [74].

## Abbreviations

DH: Department of Health; DFID: Department for International Development; EU: European Union; FCO: Foreign and Commonwealth Office; GHD: Global health diplomacy; HiFP: Health in foreign policy; HPA: Health Protection Agency; MDGs: Millennium Development Goals; NGO: Non-governmental organization; WHO: World Health Organization

## Competing interests

The authors declare that they have no competing interests.

## Authors’ contributions

MLG designed and carried out the study as part of her doctoral work under RL supervision. MLG wrote the first draft of this article. RL commented on and revised subsequent drafts. Both authors have read and agreed upon the final version.

## Authors’ information

^1^Institute of Population Health, University of Ottawa, Canada. ^2^Canada Research Chair, Globalization / Health Equity, Professor, Faculty of Medicine, Institute of Population Health, University of Ottawa, Canada.
